# Eleven ancestral gene families lost in mammals and vertebrates while otherwise universally conserved in animals

**DOI:** 10.1186/1471-2148-6-5

**Published:** 2006-01-18

**Authors:** Etienne GJ Danchin, Philippe Gouret, Pierre Pontarotti

**Affiliations:** 1 Phylogenomics Laboratory, EA 3781 EGEE, Universite de Provence, Marseilles, France; 2 Glycogenomics and Biomedical Strucural Biology, AFMB, UMR 6098 CNRS/Université de Provence/Université de la Méditerrannée, Marseilles, France

## Abstract

**Background:**

Gene losses played a role which may have been as important as gene and genome duplications and rearrangements, in modelling today species' genomes from a common ancestral set of genes. The set and diversity of protein-coding genes in a species has direct output at the functional level. While gene losses have been reported in all the major lineages of the metazoan tree of life, none have proposed a focus on specific losses in the vertebrates and mammals lineages. In contrast, genes lost in protostomes (i.e. arthropods and nematodes) but still present in vertebrates have been reported and extensively detailed. This probable over-anthropocentric way of comparing genomes does not consider as an important phenomena, gene losses in species that are usually described as "higher". However reporting universally conserved genes throughout evolution that have recently been lost in vertebrates and mammals could reveal interesting features about the evolution of our genome, particularly if these losses can be related to losses of capability.

**Results:**

We report 11 gene families conserved throughout eukaryotes from yeasts (such as *Saccharomyces cerevisiae*) to bilaterian animals (such as *Drosophila melanogaster *or *Caenorhabditis elegans*). This evolutionarily wide conservation suggests they were present in the last common ancestors of fungi and metazoan animals. None of these 11 gene families are found in human nor mouse genomes, and their absence generally extends to all vertebrates. A total of 8 out of these 11 gene families have orthologs in plants, suggesting they were present in the Last Eukaryotic Common Ancestor (LECA). We investigated known functional information for these 11 gene families. This allowed us to correlate some of the lost gene families to loss of capabilities.

**Conclusion:**

Mammalian and vertebrate genomes lost evolutionary conserved ancestral genes that are probably otherwise not dispensable in eukaryotes. Hence, the human genome, which is generally viewed as being the result of increased complexity and gene-content, has also evolved through simplification and gene losses. This acknowledgement confirms, as already suggested, that the genome of our far ancestor was probably more complex than ever considered.

## Background

It is widely acknowledged that gene losses occurred in various different lineages of the tree of life and contributed in modeling modern genomes. Reports of gene losses including losses of ancestral genes in the human genome, predate the genomic era [1,2] and generally described one to a few genes. The availability of the complete genomes and proteomes of several species including eukaryotes allowed to investigate gene losses at a larger scale [3-7], to consider gene loss as a global phenomena, and to study co-losses in different lineages. In addition, these genome-scale analyses allowed an evaluation of the ancestral proteome size at various different nodes of the tree of life, and thus provided the capacity to deduce differential losses in each daughter branches. Most "gene loss" reports and analyses [3,6,8,9] arrive at the conclusion that protostomes (like *Drosophila melanogaster *or *Caenorhabditis elegans*) underwent significantly higher rates of loss of ancestral genes than vertebrates (like humans). Krylov et al., for example, found that the human genome underwent losses of 158 gene families in comparison to the last common ancestor of all coelomates, while Drosophila lost 480 of these ancestral families [6]. Hughes et al., found that compared to the last vertebrates common ancestor, humans lost 78 gene families while fugu lost 122 of them [3]. Therefore, in a naturally anthropocentric manner, most of the analyses concentrated on the message that vertebrates, mammalian and in particular the human genome evolved through keeping more complexity in comparison to "basal phyla". The studies thus provided more emphasis on ancestral genes that were lost outside of vertebrate lineage and, more precisely on genes still present in humans but lost in other species.

However, more recent publications, such as the analysis of the chicken genome [10], reported gene families that were probably present in the ancestor of all eukaryotes but were lost in mammalian genomes as well as in other lineages. Alternatively, several authors have reported specific losses in hominoids or human lineages of genes that are conserved in all other mammals [1,2,11,12], or in all other chordates [13].

**Figure 1:**
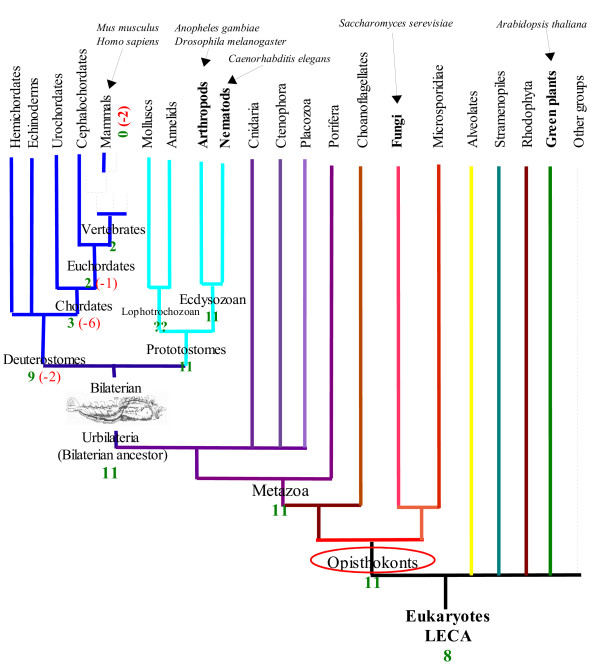
**Phylogeny of Eukaryotes with gene loss pattern**. Taxa for which a species whose genome is fully sequenced and which were used to determine clusters of ancestrally present genes lost in mammals are represented in Bold. Model species used are indicated in italics. Number of ancestral Opisthokonts gene families present at each node is indicated in green, and the number of gene families that have been lost is represented in red. Due to lack of genomic data we were unable to evaluate the number of ancestral genes still present in the Lophotrochozoan ancestor. The phylogeny represented is according to the Ecdysozoa hypothesis for the position of *Caenorhabditis elegans*, note that under the Coelomata hypothesis the loss pattern would be unchanged.

In the present analysis, we investigate the function of 11 gene families that are conserved across four model Opisthokonts species (fungi + metazoan); *Saccharomyces cerevisiae*, *Anopheles gambiae*, *Drosophila melanogaster*, and *Caenorhabditis elegans *but that are absent from *Homo sapiens *and *Mus musculus *genomes (Figure 1). Throughout the manuscript we use the term "gene-family" to describe families of homologous genes that are present as multiple copies in at least one of the species in which they are still found. The major novelty in our analysis is that we have focused on gene families that are still evolutionarily conserved at least in all the 4 Opisthokonts model species and hence are probably universally indispensable in this lineage. However as no member of these gene families are found neither in *Homo sapiens *nor in *Mus musculus*, they represent gene families lost, at least, in mammals (and possibly in other lineages). Mammalian gene losses reported so far, in contrast, have generally considered gene families that were lost multiple times and independently in several different eukaryotic lineages thus constituting non mammalian-specific losses and repeatedly dispensable genes. Since the otherwise universal conservation of the 11 gene families we identified may reveal functionally important and central genes for eukaryotes, we retrieved publicly available function for these genes. We next attempted to correlate these gene families' losses to loss of capabilities in our lineage as well as more generally in vertebrates' lineage.

At a methodological level, all genome-scale analyses published thus far are based on multiple-way best reciprocal blast hit approaches [14] to detect potential clusters of orthologous genes. Moreover, these studies were all bound to the analysis of only 7 genomes with the possibility that their annotation contain errors or missing genes. Thus, data from other species was not taken into account to complete the biodiversity coverage. The difference in our analysis is that we added two crucial steps: a phylogenetic confirmation followed by an in-depth similarity search to gain more support and evidence. Systematic phylogenetic analysis allowed correcting false-orthology inference due to multidirectional best blast hits approaches. In-depth similarity search allowed confirming loss and to provide more detailed resolution of the loss timing of genes, by exploiting all the biodiversity available in biological databases. This double confirmation turned out to eliminate a non-negligible proportion of potential clusters and putative lost gene families. This result suggest that while at a large scale automatic clusters of orthologous genes certainly provide a good overview of the general trends in comparative proteomics, careful confirmation should be considered when analyzing in detail each cluster at a small scale.

## Results and discussion

### Evolutionarily conserved genes, lost in mammals and vertebrates

By parsing clusters of orthologous eukaryotic genes, we first identified 24 groups of orthologs that have representatives in all 4 model Opisthokonts species (*Caenorhabditis elegans*, *Anopheles gambiae*, *Drosophila melanogaster*, *Saccharomyces cerevisiae*) but none neither in *Homo sapiens *nor in *Mus musculus *(see methods for more details). Using this criterion, we aimed at selecting genes that were present in the last common ancestor of Opisthokonts (Figure 1) but were specifically lost, at least, before the mammals' radiation. We performed a systematic phylogenetic analysis of all of these 24 groups followed by an in-depth BLAST similarity search (including ESTs, HTGS, WGS, and BAC databases). Phylogenetic analysis allowed the confirmation of homology relationships between the genes inside a given group. It also permitted us to refine the loss temporal pattern by placing additional species on the phylogenetic trees. The "in-depth" similarity search allowed us to check for possible traces of these gene family members in other species and to support the hypothesis of actual loss in mouse and human genomes. Only half of the 24 groups had both phylogenetically confirmed homology relationships and a total absence of trace of conservation in mouse and human genomes. Hence, our analysis, which is based upon the systematic phylogenetic analysis of pre-built clusters of orthologous genes followed by in depth verification of absence of trace of conservation, revealed that in our case, 50% of clusters of orthologous genes determined by multidirectional reciprocal best hits led to false putative gene losses. It should also be pointed out that while reciprocal blast hits' false positives can be detected and eliminated through phylogenetic analysis, false negative could also occur from such approach. Indeed, true orthologs having more complex evolutionary history can be missed by reciprocal best hits and would thus not be detected using this approach. Our analysis thus represents a minimal set of ancestral genes that were lost in human and mouse and suggests that more gene families universally conserved in eukaryotes may have recently been lost in mammals.

The 11 gene families passing both the phylogenetic analysis and the similarity search are listed on Table 1; all the other groups were either artifact due to protein sequences missing in databases or due to the pairwise method for building the predicted clusters of orthologous genes (one eliminated family was a gypsy element some remnants of which have recently been reported in the human genome [15]). It should be noted that we required very stringent parameters for parsing the groups of orthologous genes. We required that the genes be still present in all the 4 model Opisthokonts genomes (*Caenorhabditis elegans*, *Drosophila melanogaster*, *Anopheles gambiae*, and *Saccharomyces cerevisiae*); and are absent both from human and mouse genomes. We thus did not intend to estimate the total number of genes that were present in the last common ancestor of Opisthokonts, since we did not consider genes that may have been lost independently and multiple times in different lineages. Rather we focused on ancestral genes universally conserved, still present in today model metazoan species and presumably lost in mammals. The 11 gene families we identified were certainly present in the last common ancestor of fungi and metazoan animals, which is believed to have lived around one billion years ago. Interestingly, 8 of these gene families have corresponding orthologs in *Arabidopsis thaliana *and other green plants, which suggests that these gene families were present even more ancestrally in the last eukaryotic common ancestor (LECA).

**Table 1:** The twelve families of universally conserved genes missing in mammals

Gene Family Name^1^	A.t^2^	S.c^2^	A.g^2^	D.m^2^	C.e^2^	Loss extends to	Function	EC	KEGG pathway
ACH	0	1	1	1	1	Deutero.	Acetyl-CoA Hydrolase, Pyruvate Metabolism	3.1.2.1	sce00620
TPS	4	1	1	1	2	Deutero.	Trehalose 6P biosynthesis	2.4.1.15	Sce00500
YD56	4	3	2	1	1	Chordates	Multicopper Ion transporter	1.-.-.-	XXX
YMT1	1	1	1	3	1	Chordates	Putative oxidroeductase and K+ ion transporter	XXX	XXX
PNC1	0	1	1	1	1	Chordates	Nicotinate and Nicotinamide Metabolism	3.5.1.19	sce00760
YM74	3	2	1	1	1	Chordates	Transcription factor activity/DNA Binding	XXX	XXX
THDH1/ILV1	1	1	1	1	2	Chordates	Leucine, Isoleucine, Valine Biosynthesis. Threonine Metabolism	4.3.1.19	sce00260
GLT	1	1	1	1	1	Chordates	Glutamate synthesis	1.4.1.13	sce00251 sce00910
AMT/MEP -1,2,3	6	3	1	1	4	Eu-chordates	Amonium Transporter	XXX	XXX
YKH1	0	1	1	1	9	Mammals	Metabolism, oxidoreductase	XXX	XXX
URH1	2	1	3	3	2	Mammals	Uridine catabolism, hydrolase activity	3.2.2.3	XXX

^1 ^Gene family name is given according to the gene's name in *Saccharomyces cerevisiae*.

^2 ^Number of gene copies from the considered family present in the genome, for each model species, A.t = *Arabidopsis thaliana*, S.c = *Saccharomyces cerevisiae*, A.g = *Anopheles gambiae*, D.m = *Drosophila melanogaster*, C.e = *Caenorhabditis elegans*.

^3 ^KEGG's biochemical pathway's accession number for *Saccharomyces cerevisiae*.

### Phylogenetic loss pattern

Phylogenetic analyses coupled with in depth similarity search revealed that all the genes that are absent from human and mouse genomes had no trace of conservation with any mammalian sequence in protein databases and hence may reflect losses extending to all mammals. Furthermore, 9 out of the 11 groups of orthologous genes presented no trace of conservation in any vertebrate species indicating that these families were lost more ancestrally and even before the vertebrate's radiation. Only one group had orthologous counterparts in urochordates, suggesting that all the other families may have been lost at least as far back as the chordates radiation (Table 1). We propose that the 11 gene families we identified were at least present in the last common ancestor of Opisthokonts. None of these 11 gene families exhibit conservation in any mammalian species and they thus constitute at least mammalian-lineage's gene losses. The phylogenetic analysis shows that the loss pattern of the 11 gene families is different and some genes have been lost more ancestrally than others. However, as all these gene families are conserved in all Opisthokonts lineages except in chordates, they may represent functionally crucial genes for the majority of Eukaryotic life forms.

### Functional aspects of lost genes

We investigated available functional information related to the 11 gene families and tried to correlate functional data to losses of functionality or changes in environmental conditions for the species in which they are missing (Table 1). We searched various "function-centered" databases, including pathway and domain databases (more details are available on Methods). A total of 10 gene families had publicly available functional information assigned. More than 50% of the universally conserved gene families lost in mammals are involved in biomolecular metabolism/catabolism; three gene families were annotated as oxydoreductive and ion transporters, and one family as being a transcription factor (Table 1).

Interestingly, some cases of gene loss could be related to losses of functionality in vertebrates and mammals, as for example the cases of TPS1 and ILV1 gene families which we detail hereafter.

### The TPS gene family encoding Trehalose 6P synthase

**Figure 2:**
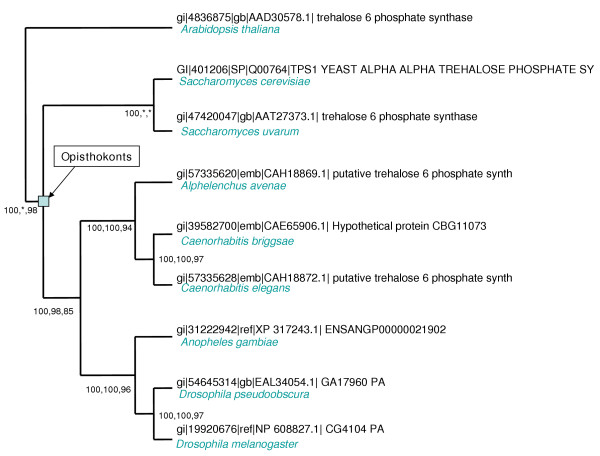
**phylogenetic tree of the TPS family**. The tree clearly shows that all the genes represented here are orthologous. Bootstrap values are indicated to evaluate each node's robustness, for Neighbor Joining, Maximum Parsimony and Maximum Likelihood methods respectively. The Opisthokonts group is represented and total absence of deuterostomian species is remarkable. This tree, as all the trees in the present analysis, was constructed with the automated phylogenomic annotation pipeline available in the FIGENIX platform [24]. For a matter of clarity, only one representative and one close relative of each of the 4 core metazoan species selected for constructing the COGS are represented on this tree. The whole tree, available upon request, includes several other fungal, plant and protostomian species.

This family is found in plants, fungi, arthropods, and nematodes but appears to be missing in mammals and more widely in vertebrates' genomes (Figure 2). This gene family loss can be correlated to the loss of the ability to biosynthesize Trehalose 6P from UDP-glucose. This disaccharide, which works as a storage carbohydrate, is crucial for the survival of species in dry and freezing periods and under other stress conditions [16]. Its presence in bacteria, in addition to Opisthokonts lineages suggests it was ancestrally present on the earliest lifeforms on earth. Mammals are unable to produce Trehalose 6P, and actually Trehalose 6P is not found in those species [17]. Moreover, Guo et al. [18] ectopically expressed bacterial Trehalose biosynthesis genes in human cells and showed that these cells acquired tolerance to desiccation. Trehalose biosynthesis from UDP glucose requires two enzymes, TPS (trehalose 6P synthase, EC:2.4.1.15), and TPP (trehalose 6P phosphatase, EC:3.1.3.12), this information allowed us to identify a 13^th ^group of lost genes in human and mouse. Indeed, no trace of conservation could be found in any database in mammals for TPP. This gene family is present in plants, yeasts, and arthropods, but has also been lost in nematodes [17], and for that reason, it did not pass our criterion in the parsing of groups of orthologous genes, since we required presence in all the 4 model species and did not consider multiple convergent losses.

### The THDH/ILV1 family encoding Threonine deaminases

Members of this gene family are found in plants, yeasts, arthropods, and nematodes. No trace of conservation can be found in mammals or chordates, which suggests that this family was lost before chordates' radiation. Members of this family are all involved in Threonine metabolism and biosynthesis of 2-Oxobutanoate, which is used as a precursor in the biosynthesis pathway of 3 other aminoacids: Valine, Leucine and Isoleucine. These amino acids are all essential amino acids that mammals are unable to synthesize and must be provided by the diet. It should be noted here that while arthropods and nematodes are able to biosynthesize 2-Oxobutanoate from Threonine, they are also unable to synthesize these essential aminoacids due to the loss of enzymes further down the pathway (ILV3, ILV5) as shown by Hughes et al. [4]. The universal capability (found from plants to arthropods) that we have lost is thus, in this case, the biosynthesis of 2-Oxobutanoate from Threonine.

Most of the other families lost in vertebrates and mammals could not be correlated with clear loss of functionality, either because not enough functional information was available for the considered genes or because the same functionality was replaced by other genes in mammals and vertebrates. This latter case is illustrated by the GLT family that, although present in plants, yeasts, arthropods and nematodes, is absent from all vertebrates. This family encodes enzymes involved in glutamate and nitrogen metabolism (EC:1.4.1.13) which catalyzes the reversible reaction L-Glutamate → L-Glutamine, with NADP+ or NAD+ as acceptor partner. This reaction is also possible in vertebrates though it is catalyzed by another enzyme (EC:6.3.5.4) and uses other partners (L-aspartate and L-asparagine). Interestingly, the loss pattern of these enzymes is different between the various eukaryotic lineages. Indeed, while Plants, Yeasts and Nematodes have the two enzymes and are thus able to transform L-Glutamine and L-Glutmamate using two different pathways, Arthropods only have one enzyme (EC:1.4.1.13), and vertebrates only have the other one (EC:6.3.5.4). The loss pattern is thus complex in this case. The last eukaryotic common ancestor had at least these two enzymes and one of them was lost in the vertebrate's lineage while the other one was lost in arthropods.

As a complementary analysis to mammalian and vertebrates' losses of otherwise universally present Opisthokonts genes we identified and analyzed, we also performed a survey of previously reported gene losses in mammals in the literature. We re-investigated each reported gene loss in order to confirm them (using in depth similarity search explained in Methods) and to give more details about the temporal pattern of the loss of these genes (see the detailed report on the supplementary material 1).

## Conclusion

### Gene loss and our ancestor's genomic "complexity"

We reported here an in depth analysis of a dozen of gene families that were all at least present in the last common ancestor of fungi and metazoan (Opisthokonts), are still present and conserved in all modern Opisthokonts model species, but are specifically absent from human and mouse genomes. Our analysis shows that, as with other species, the human genome and other mammalian genomes have undergone losses of gene families compared to the genome of the last common ancestor of all Opisthokonts. Mammals and more generally vertebrates thus appear to have recently and specifically lost genes that were conserved for around 1 billion years. These genes may thus constitute universally indispensable genes in all Opisthokonts except in mammals and vertebrates. Most of the lost genes we report in this analysis were already present in LECA. It thus indicates that mammalian and vertebrates' lineages have lost genes that were more ancestrally present in the earliest lifeforms on earth and are still widely present and conserved in other eukaryotic lineages. The total number of such genes we have lost is certainly higher. We reported here the existence of at least 11 families of such genes and correlated the losses with functional data. In addition to confirming that the human genome also evolved through gene losses of very ancestral genes and not only by duplication events and the emergence of "new genes", this study also reinforces the hypothesis first formulated by Kortschak et al [8], that the genome of our far ancestor was more complex than usually considered.

## Methods

**Figure 3:**
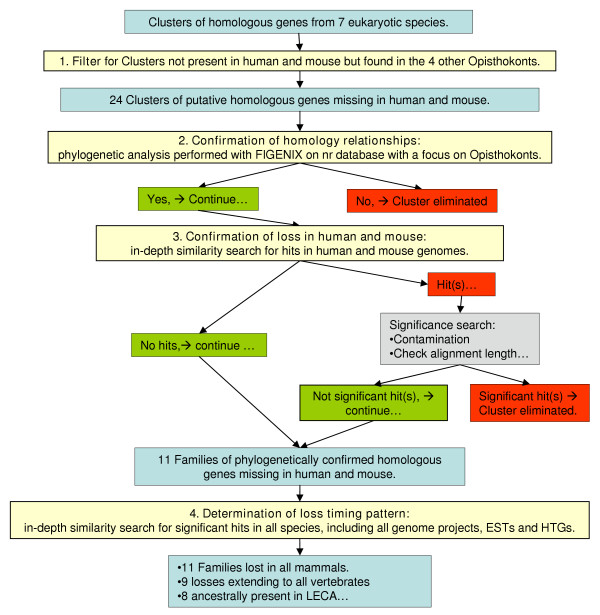
**general strategy used for identifying lineage-specific gene family losses**. This chart represents the general strategy we adopted to find gene-families that were specifically lost in specific lineages of the tree of life. The 4 main steps are represented in yellow boxes, main results and data in blue boxes, and decisions in red and green boxes.

The general strategy adopted to identify putative families of homologous genes lost in mammals and vertebrates is schematized on figure 3.

### Selection of putative universally conserved gene families lost in mammals

Zdobnov et al. constructed clusters of putative orthologous genes in their comparative genomic analysis of the Drosophila and Anopheles genomes [19]. These clusters resemble the COGS and KOGS performed by Koonin et al [20,21] as they were also built based on multiple way best reciprocal blast hits. The COGs from Zdobnov et al. included 7 eukaryotic species (*Caenorhabditis elegans*, *Drosophila melanogaster*, *Anopheles gambiae*, *Arabidopsis thaliana*, *Saccharomyces cerevisiae*, *Homo sapiens*, and *Mus musculus*) whose genome and proteome annotation were available. The complete dataset containing all the clusters of orthologous groups was kindly provided by Dr. Christian von Mering. Using UNIX grep and awk filters, we selected clusters of putative orthologous genes containing representatives from all the 4 following species: *Caenorhabditis elegans*, *Drosophila melanogaster*, *Anopheles gambiae*, *Saccharomyces cerevisiae*, but in which *Homo sapiens *and *Mus musculus *were missing. We thus selected genes likely to have been ancestrally present in the last common ancestor of metazoan animals and fungi (the Opisthokonts ancestor), but that were lost at least in the human and mouse genomes. We chose to focus on genes lost at least in mammals rather than human specific losses, because double losses (in human and mouse) reinforce the hypothesis of an actual loss and minimize the risk of artifact due to incomplete databases or annotation errors.

### Confirmation of the orthology and paralogy relationships inside the putative COGs

To filter false orthology and paralogy relationships determined by multidirectional reciprocal best blast hit analyses such as the one used in the construction of these COGS, we performed a systematic phylogenetic analysis of each cluster. Indeed, reciprocal gene losses as well as genes actually missing in protein databases lead to false orthology and paralogy relationships, thus producing artificial COGs [22,23]. These false relationships can only be detected and resolved by in-depth phylogenetic analysis including additional taxa and comparison with a reference tree of life [24]. To perform the phylogenetic analysis, we used the phylogenomic analysis pipeline available in FIGENIX automated genomic annotation platform [24]. This pipeline uses as input any protein sequence and can be summarized in the following steps (the whole pipeline runs around 50 different steps):

- Produces blast similarity search.

- Filters blast results for significant similarity.

- Produces a multiple sequence alignment (MSA).

- Eliminates from the MSA sequences and columns producing potential biases in the phylogenetic reconstruction.

- Reconstructs phylogenetic trees using three different methods, Maximum Parsimony [25], Maximum Likelihood [26] and Neighbor Joining [27].

- Builds a consensus of the three trees and reports bootstrap values given by each method.

- Compares the consensus tree to a reference species tree of life.

- Deduces orthology and paralogy relationships.

For each putative group of orthologous genes, we chose the Yeast protein as query sequence, for blast searches we selected the NCBI nr protein database and we used the NCBI taxonomic tree as the reference tree of life. All orthology and paralogy relationships were thus phylogenetically verified and false-positive COGs were eliminated from the dataset.

### Determining the loss temporal pattern

Detailed phylogenetic analysis coupled with in depth similarity-search of all the confirmed clusters of orthologous genes allowed, apart from confirming homology relationships, to check to what extent genes, orthologous to the genes of the considered family are found in other species. Phylogenetic trees for each family are available upon request.

### Supporting the loss hypothesis in mammals and vertebrates

For each phylogenetically confirmed cluster of orthologous genes in which at least *Caenorhabditis elegans*, *Drosophila melanogaster*, *Anopheles gambiae*, *Saccharomyces cerevisiae *are altogether present; and human and mouse are both absent; we sought in depth for traces of conservation in mouse and human genomes, and more generally in mammalian and vertebrates genomes using blast. We performed blastp searches with relaxed stringency on Swissprot, NR, and Ensembl proteomes. If no hit was found we performed a further tblastn analysis with relaxed stringency on complete genomes, HTGS, ESTs, Traces Repositories, and WGS databases. In cases where there are still no hit, and for species having their genome completely sequenced as human and mouse, the most likely hypothesis is that the gene family has actually been lost in the corresponding species. Multiplying the number of mammalian and vertebrate genomes searched against reinforces the hypothesis of an actual loss, and in the present analysis we used all publicly available genomic information.

### Functional information mining

For each cluster of orthologous genes lost in mammals, we retrieved available functional information related to the genes in *Caenorhabditis elegans*, *Drosophila melanogaster*, *Anopheles gambiae*, *Saccharomyces cerevisiae*, and *Arabidopsis thaliana *(when green plants' orthologs were present). We searched the KEGG [28] database for known pathways in which the genes are involved, and known enzymatic properties and reactions in which they are involved (EC codes). We searched flybase, Wormbase, MGI, SGD, TAIR, and Swissprot databases for annotated functional information (Biochemical Function, Biological Process, and Cellular Component) in the gene ontology format [29]. We also used Interpro and Pfam [30] web servers to retrieve protein domains information for all these species.

## Authors' contributions

EGJD wrote the manuscript and designed and performed the whole analysis. PG participated in designing UNIX awk and grep scripts for parsing results of the clusters of orthologous genes. PP supervised the whole analysis and participated in analyzing the results and in drawing conclusions.

## Supplementary Material

Additional File 1 Literature report of other vertebrates and mammals specific gene losses. supplementary materials describe the results of our literature mining of previously reported gene loss in vertebrates and mammals. It also highlights the difference with the losses we report here and also reports more recent human-specific losses. Click here for file

## References

[B1] Matsumoto M, Nomura T, Momose K, Ikeda Y, Kondou Y, Akiho H, Togami J, Kimura Y, Okada M, Yamaguchi T (1996). Inactivation of a novel neuropeptide Y/peptide YY receptor gene in primate species. J Biol Chem.

[B2] Haag F, Koch-Nolte F, Kuhl M, Lorenzen S, Thiele HG (1994). Premature stop codons inactivate the RT6 genes of the human and chimpanzee species. J Mol Biol.

[B3] Hughes AL, Friedman R (2004). Differential loss of ancestral gene families as a source of genomic divergence in animals. P Roy Soc Lond B Bio.

[B4] Hughes AL, Friedman R (2004). Shedding genomic ballast: Extensive parallel loss of ancestral gene families in animals. J Mol Evol.

[B5] Koonin EV, Fedorova ND, Jackson JD, Jacobs AR, Krylov DM, Makarova KS, Mazumder R, Mekhedov SL, Nikolskaya AN, Rao BS, Rogozin IB, Smirnov S, Sorokin AV, Sverdlov AV, Vasudevan S, Wolf YI, Yin JJ, Natale DA (2004). A comprehensive evolutionary classification of proteins encoded in complete eukaryotic genomes. Genome Biol.

[B6] Krylov DM, Wolf YI, Rogozin IB, Koonin EV (2003). Gene loss, protein sequence divergence, gene dispensability, expression level, and interactivity are correlated in eukaryotic evolution. Genome Res.

[B7] Bertrand S, Brunet FG, Escriva H, Parmentier G, Laudet V, Robinson-Rechavi M (2004). Evolutionary Genomics of Nuclear Receptors: From 25 Ancestral Genes to Derived Endocrine Systems. Mol Biol Evol.

[B8] Kortschak RD, Samuel G, Saint R, Miller DJ (2003). EST analysis of the cnidarian Acropora millepora reveals extensive gene loss and rapid sequence divergence in the model invertebrates. Curr Biol.

[B9] Raible F, Arendt D (2004). Metazoan evolution: Some animals are more equal than others. Curr Biol.

[B10] Hillier LW, Miller W, Birney E, Warren W, Hardison RC, Ponting CP, Bork P, Burt DW, Groenen MA, Delany ME, Dodgson JB, Chinwalla AT, Cliften PF, Clifton SW, Delehaunty KD, Fronick C, Fulton RS, Graves TA, Kremitzki C, Layman D, Magrini V, McPherson JD, Miner TL, Minx P, Nash WE, Nhan MN, Nelson JO, Oddy LG, Pohl CS, Randall-Maher J, Smith SM, Wallis JW, Yang SP, Romanov MN, Rondelli CM, Paton B, Smith J, Morrice D, Daniels L, Tempest HG, Robertson L, Masabanda JS, Griffin DK, Vignal A, Fillon V, Jacobbson L, Kerje S, Andersson L, Crooijmans RP, Aerts J, van der Poel JJ, Ellegren H, Caldwell RB, Hubbard SJ, Grafham DV, Kierzek AM, McLaren SR, Overton IM, Arakawa H, Beattie KJ, Bezzubov Y, Boardman PE, Bonfield JK, Croning MD, Davies RM, Francis MD, Humphray SJ, Scott CE, Taylor RG, Tickle C, Brown WR, Rogers J, Buerstedde JM, Wilson SA, Stubbs L, Ovcharenko I, Gordon L, Lucas S, Miller MM, Inoko H, Shiina T, Kaufman J, Salomonsen J, Skjoedt K, Wong GK, Wang J, Liu B, Yu J, Yang H, Nefedov M, Koriabine M, Dejong PJ, Goodstadt L, Webber C, Dickens NJ, Letunic I, Suyama M, Torrents D, von Mering C, Zdobnov EM, Makova K, Nekrutenko A, Elnitski L, Eswara P, King DC, Yang S, Tyekucheva S, Radakrishnan A, Harris RS, Chiaromonte F, Taylor J, He J, Rijnkels M, Griffiths-Jones S, Ureta-Vidal A, Hoffman MM, Severin J, Searle SM, Law AS, Speed D, Waddington D, Cheng Z, Tuzun E, Eichler E, Bao Z, Flicek P, Shteynberg DD, Brent MR, Bye JM, Huckle EJ, Chatterji S, Dewey C, Pachter L, Kouranov A, Mourelatos Z, Hatzigeorgiou AG, Paterson AH, Ivarie R, Brandstrom M, Axelsson E, Backstrom N, Berlin S, Webster MT, Pourquie O, Reymond A, Ucla C, Antonarakis SE, Long M, Emerson JJ, Betran E, Dupanloup I, Kaessmann H, Hinrichs AS, Bejerano G, Furey TS, Harte RA, Raney B, Siepel A, Kent WJ, Haussler D, Eyras E, Castelo R, Abril JF, Castellano S, Camara F, Parra G, Guigo R, Bourque G, Tesler G, Pevzner PA, Smit A, Fulton LA, Mardis ER, Wilson RK (2004). Sequence and comparative analysis of the chicken genome provide unique perspectives on vertebrate evolution. Nature.

[B11] Varki A (2001). Loss of N-glycolylneuraminic acid in humans: Mechanisms, consequences, and implications for hominid evolution. Am J Phys Anthropol.

[B12] IHGSC (2004). Finishing the euchromatic sequence of the human genome. Nature.

[B13] Winter H, Langbein L, Krawczak M, Cooper DN, Jave-Suarez LF, Rogers MA, Praetzel S, Heidt PJ, Schweizer J (2001). Human type I keratin pseudogene phi hHaA has functional orthologs in the chimpanzee and gorilla: evidence for recent inactivation of the human gene after the Pan-Homo divergence. Human Genetics.

[B14] Altschul SF, Madden TL, Schaffer AA, Zhang J, Zhang Z, Miller W, Lipman DJ (1997). Gapped BLAST and PSI-BLAST: a new generation of protein database search programs. Nucleic Acids Res.

[B15] Youngson NA, Kocialkowski S, Peel N, Ferguson-Smith AC (2005). A small family of sushi-class retrotransposon-derived genes in mammals and their relation to genomic imprinting. J Mol Evol.

[B16] Eastmond PJ, Graham IA (2003). Trehalose metabolism: a regulatory role for trehalose-6-phosphate?. Curr Opin Plant Biol.

[B17] Pellerone FI, Archer SK, Behm CA, Grant WN, Lacey MJ, Somerville AC (2003). Trehalose metabolism genes in Caenorhabditis elegans and filarial nematodes. Int J Parasitol.

[B18] Guo N, Puhlev I, Brown DR, Mansbridge J, Levine F (2000). Trehalose expression confers desiccation tolerance on human cells. Nat Biotechnol.

[B19] Zdobnov EM, von Mering C, Letunic I, Torrents D, Suyama M, Copley RR, Christophides GK, Thomasova D, Holt RA, Subramanian GM, Mueller HM, Dimopoulos G, Law JH, Wells MA, Birney E, Charlab R, Halpern AL, Kokoza E, Kraft CL, Lai Z, Lewis S, Louis C, Barillas-Mury C, Nusskern D, Rubin GM, Salzberg SL, Sutton GG, Topalis P, Wides R, Wincker P, Yandell M, Collins FH, Ribeiro J, Gelbart WM, Kafatos FC, Bork P (2002). Comparative genome and proteome analysis of Anopheles gambiae and Drosophila melanogaster. Science.

[B20] Tatusov RL, Galperin MY, Natale DA, Koonin EV (2000). The COG database: a tool for genome-scale analysis of protein functions and evolution. Nucleic Acids Res.

[B21] Tatusov RL, Fedorova ND, Jackson JD, Jacobs AR, Kiryutin B, Koonin EV, Krylov DM, Mazumder R, Mekhedov SL, Nikolskaya AN, Rao BS, Smirnov S, Sverdlov AV, Vasudevan S, Wolf YI, Yin JJ, Natale DA (2003). The COG database: an updated version includes eukaryotes. BMC Bioinformatics.

[B22] Jordan IK, Wolf YI, Koonin EV (2004). Duplicated genes evolve slower than singletons despite the initial rate increase. BMC Evol Biol.

[B23] Danchin EGJ (2004). Reconstruction of ancestral genomic regions by comparative analysis of evolutionary conserved syntenies. Towards reconstructing the genome of the ancestor of all Bilaterian species (Urbilateria).. Bioinformatics, Structural biochemistry, Genomics.

[B24] Gouret P, Vitiello V, Balandraud N, Gilles A, Pontarotti P, Danchin EGJ (2005). FIGENIX: Intelligent automation of genomic annotation: expertise integration in a new software platform. BMC Bioinformatics.

[B25] Fitch WM (1971). Toward defining the course of evolution: Minimum change for a specific tree topology.. Systematic Zoology.

[B26] Felsenstein J (1981). Evolutionary trees from DNA sequences: a maximum likelihood approach. J Mol Evol.

[B27] Saitou N, Nei M (1987). The neighbor-joining method: a new method for reconstructing phylogenetic trees. Mol Biol Evol.

[B28] Kanehisa M, Goto S, Kawashima S, Okuno Y, Hattori M (2004). The KEGG resource for deciphering the genome. Nucleic Acids Res.

[B29] Ashburner M, Ball CA, Blake JA, Botstein D, Butler H, Cherry JM, Davis AP, Dolinski K, Dwight SS, Eppig JT, Harris MA, Hill DP, Issel-Tarver L, Kasarskis A, Lewis S, Matese JC, Richardson JE, Ringwald M, Rubin GM, Sherlock G (2000). Gene ontology: tool for the unification of biology. The Gene Ontology Consortium. Nat Genet.

[B30] Bateman A, Coin L, Durbin R, Finn RD, Hollich V, Griffiths-Jones S, Khanna A, Marshall M, Moxon S, Sonnhammer EL, Studholme DJ, Yeats C, Eddy SR (2004). The Pfam protein families database. Nucleic Acids Res.

