# Supplementary material for: Eleven ancestral gene families lost in mammals and vertebrates while otherwise universally conserved in animals
Source: BMC Evol Biol. 2006 Jan 18;6:5. doi: 10.1186/1471-2148-6-5 (PMC1382263; doi:10.1186/1471-2148-6-5)
Supplement: Additional File 1 — Literature report of other vertebrates and mammals specific gene losses. supplementary materials describe the results of our literature mining of previously reported gene loss in vertebrates and mammals. It also highlights the difference with the losses we report here and also reports more recent human-specific losses. [file 1471-2148-6-5-S1.pdf]

Etienne G.J. Danchin, Philippe Gouret, Pierre Pontarotti.

Supporting Online Material

As a complementary analysis to mammalian and vertebrates' losses of otherwise universally present Opisthokonts gene families we analyzed; we also performed a survey of previously reported gene losses in mammals in the literature. We re-investigated each reported gene loss in order to confirm them (using in depth similarity search explained in Methods) and to give more details about the temporal pattern of the loss of these genes.

Losses extending to all mammals.

In the recent analysis of the chicken genome (Hillier et al. 2004), the authors identified several genes that have been lost in mammals in comparison to the last common ancestor of vertebrates. Some of these genes are found outside the vertebrates' lineage suggesting they were already present in farther common ancestors. Nevertheless, none of these genes --contrary to those of our analysis--are still conserved in all the eukaryotic model species. Hereafter, we give details on the timing of these gene losses and their pattern of presence / absence in the tree of life.

Avidines: present in Chicken, Turkey, Echinoderms and Bacteria suggesting the gene was present in LECA, the gene is missing (in addition to mammals), in arthropods, nematodes, yeasts, and plants. The gene may thus have undergone successive convergent losses in various different lineages during eukaryotes evolution.

Vitellogenines: the gene is widely found in bilaterian species suggesting it was present in the last common ancestor of all bilaterian (Urbilateria). In addition to mammals, the gene

appears to have also been lost in Drosophila and no clear significant similarity is found outside bilaterian.

CPD Photolyase: the gene is found in marsupials suggesting the loss is specific to placental mammals. Arthropods and Plants appear to have orthologous counterpart of this gene while in contrast yeasts and nematodes present no trace of conservation with this gene. We can thus hypothesize that this gene was ancestrally present in LECA and was secondary lost several times in various different eukaryotic lineages.

Indigoidine: chicken and other vertebrates, as well as arthropods nematodes and yeasts have this gene while it is lost specifically in mammals. It should be noted that this mammalian specific loss of Opisthokont's ancestrally present gene was not detected by our filter because the gene is missing in S.cerevisiae while present in S.pombe. It thus confirms that the total number of gene families that were present in the last common ancestor of Opisthokonts, and were recently lost in mammals is certainly higher.

Genes specifically lost in the human or hominidae lineages.

In the present analysis we focused on genes that were lost at least at the basis of mammalian lineages and were ancestrally present at the basis of the Opisthokonts (Fungi + Metazoa) lineage and for some of them in the last Eukaryotic Common Ancestor (LECA). Genes that were more recently lost in a specific manner in H.sapiens could also be investigated. For reliability reasons we have not reported in our analysis genes ancestrally present but lost specifically in humans or hominidae because we required that the loss was supported by genes missing in at least two species whose genomes are completely sequenced (i.e. Mouse and Human). However, specific losses in humans or hominidae have

been reported in the literature. Several categories of losses can be distinguished as a function of the ancestral presence of the gene families. In the analysis of the latest human genome assembly (IHGSC 2004), the authors identify 32 recent pseudogenes in our genome corresponding to active genes in mouse and rat genomes. A total of 27 out of these 32, are also pseudogenes in chimpanzee suggesting these genes have been inactivated in the hominidae lineage. In contrast 5 out of the 32 human pseudogenes have active counterpart in chimpanzee and may thus represent specific losses in the human genome.

Varki et al (Varki 2001), reported the specific loss of N-glycolylneuraminic acid gene in humans while this gene is shared by all other mammals. Haag et al (Haag et al. 1994) identified a gene (RT6) which is also widely conserved in mammals but lost both in Human and Chimpanzee and probably all hominidae, while Matsumoto et al (Matsumoto et al. 1996) identified a gene (encoding a neuropeptide Y/peptide YY receptor) also specific to mammals but lost in all primates.

A gene encoding for Myosin Heavy Chain, expressed in masticatory muscles of most of primates, was reported to be specifically lost in the Homo lineage by Stedman et al. (Stedman et al. 2004).

Concerning specific losses in humans of genes more ancestrally present, it should be noted that Winter et al (Winter et al. 2001) identified a gene (KRTHAP1) shared between all chordates species but missing in the homo lineage. Oda et al. (Oda et al. 2002) identified an even more ancestral gene widely present in eukaryotic species but specifically and recently lost in human and great apes.

References

Haag, F., F. Koch-Nolte, M. Kuhl, S. Lorenzen, and H. G. Thiele. 1994. Premature stop codons inactivate the RT6 genes of the human and chimpanzee species. J Mol Biol **243**:537-546.

Hillier, L. W.W. MillerE. Birney et al. 2004. Sequence and comparative analysis of the chicken genome provide unique perspectives on vertebrate evolution. Nature **432**:695-716.

IHGSC. 2004. Finishing the euchromatic sequence of the human genome. Nature **431**:931-945.

Matsumoto, M., T. Nomura, K. Momose, Y. Ikeda, Y. Kondou, H. Akiho, J. Togami, Y. Kimura, M. Okada, and T. Yamaguchi. 1996. Inactivation of a novel neuropeptide Y/peptide YY receptor gene in primate species. J Biol Chem **271**:27217-27220.

Oda, M., Y. Satta, O. Takenaka, and N. Takahata. 2002. Loss of urate oxidase activity in hominoids and its evolutionary implications. Mol Biol Evol **19**:640-653.

Stedman, H. H., B. W. Kozyak, A. Nelson, D. M. Thesier, L. T. Su, D. W. Low, C. R. Bridges, J. B. Shrager, N. Minugh-Purvis, and M. A. Mitchell. 2004. Myosin gene mutation correlates with anatomical changes in the human lineage. Nature **428**:415-418.

Varki, A. 2001. Loss of N-glycolylneuraminic acid in humans: Mechanisms, consequences, and implications for hominid evolution. Am J Phys Anthropol **Suppl 33**:54-69.

Winter, H., L. Langbein, M. Krawczak, D. N. Cooper, L. F. Jave-Suarez, M. A. Rogers, S. Praetzel, P. J. Heidt, and J. Schweizer. 2001. Human type I keratin pseudogene phi hHaA has functional orthologs in the chimpanzee and gorilla: evidence for recent inactivation of the human gene after the Pan-Homo divergence. Human Genetics **108**:37-42.
